# Emerging Role for Corticotropin Releasing Factor Signaling in the Bed Nucleus of the Stria Terminalis at the Intersection of Stress and Reward

**DOI:** 10.3389/fpsyt.2013.00042

**Published:** 2013-05-29

**Authors:** Yuval Silberman, Danny G. Winder

**Affiliations:** ^1^Neuroscience Program in Substance Abuse, Department of Molecular Physiology and Biophysics, Vanderbilt Brain Institute, Nashville, TN, USA; ^2^Kennedy Center for Research on Human Development, Vanderbilt Brain Institute, Nashville, TN, USA

**Keywords:** extended amygdala, reinstatement, relapse, excitatory transmission, addiction

## Abstract

Stress and anxiety play an important role in the development and maintenance of drug and alcohol addiction. The bed nucleus of the stria terminalis (BNST), a brain region involved in the production of long-term stress-related behaviors, plays an important role in animal models of relapse, such as reinstatement to previously extinguished drug-seeking behaviors. While a number of neurotransmitter systems have been suggested to play a role in these behaviors, recent evidence points to the neuropeptide corticotropin releasing factor (CRF) as being critically important in BNST-mediated reinstatement behaviors. Although numerous studies indicate that the BNST is a complex brain region with multiple afferent and efferent systems and a variety of cell types, there has only been limited work to determine how CRF modulates this complex neuronal system at the circuit level. Recent work from our lab and others have begun to unravel these BNST neurocircuits and explore their roles in CRF-related reinstatement behaviors. This review will examine the role of CRF signaling in drug addiction and reinstatement with an emphasis on critical neurocircuitry within the BNST that may offer new insights into treatments for addiction.

## Introduction

Alcohol and drug addiction are chronically relapsing disorders in which alcohol/drug use progresses from initial stages of limited, non-dependent intake to later stages of uncontrolled abuse (Koob, 2009; Koob and Volkow, 2010). One prominent theory posits that initial periods of use are driven primarily by the positive reinforcing value of drugs and alcohol (euphoria) while later stages of alcohol/drug addiction are driven by negative reinforcement (relief of withdrawal-induced negative affective states) (Koob and Volkow, 2010). The primary reinforcing effects of alcohol and other drugs are thought to occur by increased dopamine (DA) signaling that leads to enhanced activity of the mesocorticolimbic pathway, which in turn likely leads to escalated craving (Wise, 1980; Di Chiara and Imperato, 1988; Di Chiara, 2002; Volkow et al., 2003). Escalated alcohol/drug taking and prolonged binge episodes are thought to result in adaptation to the mesocorticolimbic pathway that results in devaluation of natural rewards, diminished cognitive control of behaviors, and increased salience of drug-related stimuli (Koob and Le, 2001; Koob and Volkow, 2010). During this time, the dorsal striatum, which typically plays a limited role in the acute reinforcing effects of drugs, becomes engaged after prolonged drug exposures and promotes compulsive drug-seeking typical in addiction (Everitt et al., 2008). For more complete reviews of mesocorticolimbic function in the initiation of drug addiction refer to (Feltenstein and See, 2008; Koob and Volkow, 2010).

Stressors and negative affective states, such as anxiety and depression, are often cited by recovering addicts as key instigators of drug craving and relapse (Sinha, 2007). Drug/alcohol binges are typically followed by various lengths of drug-withdrawal periods and numerous studies have shown that repeated binge/withdrawal episodes can recruit and sensitize brain regions associated with negative affective states, such as those that comprise the extended amygdala (for review see Koob, 2008; Koob and Volkow, 2010). Once recruited during withdrawal, brain regions associated with negative affect can remain hypersensitive even after extended periods of abstinence (Santucci et al., 2008). Furthermore, relief of negative emotional states is thought to be a critical component of alcohol/drug seeking during withdrawal (Koob, 2009). This suggests that brain regions associated with stress reactivity and negative affect, particularly the extended amygdala, become hypersensitive following repeated binge/withdrawal cycles and may mediate the transition to long-term addictive behaviors via negative reinforcement.

Altogether, these ideas support an important role of stress-related neurocircuitry in the progression of addiction and in relapse. Clinical studies on relapse have been paralleled and now extended in preclinical studies utilizing reinstatement models (Shaham et al., 2003). In this manuscript, we will review recent findings on the neurocircuitry of drug-seeking behaviors with a specific focus on those systems involved in enhanced drug-seeking during stress-induced relapse. We will also highlight potential mechanisms by which stress-related neurocircuitry may modulate drug-seeking behaviors that could be used for potential treatment targets for alcoholism and drug addiction.

## Neurocircuitry Involved in Drug Seeking during Withdrawal and Reinstatement

Reinstatement models typically involve training an animal to work to receive a drug or alcohol for a given period of time, then extinguishing that behavior before triggering the animal to seek out drugs again (Shaham et al., 2003; Epstein et al., 2006). Typical triggers of reinstatement are (1) re-exposure to the same or related drug previously administered (drug-induced reinstatement), (2) giving the animal drug-associated stimuli or cues (cue-induced reinstatement), or (3) exposure to a variety of stressors (stress-induced reinstatement). Work from reinstatement models has shown distinct roles of multiple brain regions and neurotransmitter systems in each type of reinstatement.

### Neurocircuitry of drug-induced reinstatement

A great deal of research has shown that increased activity of brain regions projecting to the mesocortical DA system is a critical factor in drug-induced reinstatement models (for review see Kalivas and Volkow, 2005; Feltenstein and See, 2008). One pathway shown to be critical to drug-induced reinstatement is a glutamatergic projection from the medial prefrontal cortex to the nucleus accumbens (Stewart and Vezina, 1988; Cornish and Kalivas, 2000; McFarland and Kalivas, 2001). Furthermore, limbic areas like the basolateral amygdala (BLA) may play a role in drug-induced reinstatement by enhanced activity of its glutamatergic projections to mesocorticolimbic system (McFarland and Kalivas, 2001; Fuchs and See, 2002). Therefore drug-induced reinstatement likely occurs via increased glutamatergic transmission to enhance mesocorticolimbic pathway activity, likely from cortical and limbic areas as well as by direct action of the drug of abuse on mesocorticolimbic DA receptors (for review see, Feltenstein and See, 2008).

### Neurocircuitry of cue-induced reinstatement

In addition to its role in drug-induced reinstatement, numerous studies have shown an important role for the BLA in cue-induced reinstatement. Exposure to drug-associated cues results in increased DA release and increased c-fos activation in the BLA following withdrawal (Neisewander et al., 1998; Weiss et al., 2000). Furthermore, intra-BLA injections of DA receptor antagonists block cue-induced reinstatement (See et al., 2001). Stimulation of the BLA has been shown to increase DA efflux in the nucleus accumbens via a glutamate receptor-dependent mechanism (Howland et al., 2002) suggesting an important role of glutamatergic afferents to the mesolimbic DA system in cue-induced reinstatement. The medial prefrontal cortex (Van den Oever et al., 2010) and the central nucleus of the amygdala (Radwanska et al., 2008) have also been shown to be important in cue-induced reinstatement.

Overall, these findings suggest that DA or glutamatergic neurotransmission in the mesocorticolimbic pathway or its afferents could be targets for therapies to reduce relapse in recovering addicts. However, use of dopaminergic agonists has yet to be proven effective for long-term relapse treatment (Lingford-Hughes et al., 2010) and may be problematic in regards to abuse liability (Shorter and Kosten, 2011). In addition, therapeutics targeting DA receptors may be problematic because of potential side effects due to interactions with motor systems or interactions with the cardiovascular system since modulating DA receptor activity can have effects on hemodynamics and cardiovascular function (Zeng et al., 2007; Banday and Lokhandwala, 2008). Furthermore, drugs targeting glutamatergic transmission given orally may also cause problematic side-effects as modulating glutamate receptors can adversely affect many other brain regions not involved in reinstatement. These findings leave the field open to the need of more selective DA or glutamatergic drugs or drugs targeting different receptor systems.

### Extended amygdala neurocircuitry in stress-induced reinstatement

Stress-induced reinstatement may be a critical model for finding suitable therapeutic targets for two important reasons. First, recovering addicts can work to modify their behavior to avoid drug re-exposure and exposure to drug-related cues as often as possible while stress in daily human life is virtually inevitable. Situations like family issues, finding and maintaining work, and even traffic in daily commutes can be stressful events to any person and may be sensitized in recovering addicts. Therefore, it is not surprising that stress is a major trigger for relapse in addicted patients (Sinha, 2007) and may make therapies targeting this system more likely to be effective in preventing relapse. Second, the neuromodulatory systems involved in stress-induced reinstatement described below may make for better pharmacotherapeutic targets due to their limited abuse liability and potentially less significant side effect profiles.

A great deal of work has examined stress-induced relapse in the preclinical setting, and a variety of stressors have been shown to reinstate drug-seeking behaviors or preference. These include footshock, restraint stress, and forced swim stress (Shaham et al., 2003; Tzschentke, 2007; Shalev et al., 2010). These studies have revealed key neurobiological mechanisms of stress-induced reinstatement, with a particular focus on the effects of two stress-related neuromodulatory systems, norepinephrine (NE) and corticotropin releasing factor (CRF), in two related brain regions of the extended amygdala, the central nucleus of the amygdala and bed nucleus of the stria terminalis (BNST) (Shaham et al., 2003; Epstein et al., 2006; Sofuoglu and Sewell, 2009; Erb, 2010; Haass-Koffler and Bartlett, 2012).

Withdrawal from chronic drug abuse can lead to NE dysfunction in the clinical population that is associated with increased vulnerability to anxiety (McDougle et al., 1994). Numerous preclinical studies have also shown drug-withdrawal-induced increases in anxiety-like behaviors and withdrawal-induced escalation in drug intake can be ameliorated by blockade of β- and α1-adrenergic receptors (ARs) (Rudoy and Van Bockstaele, 2007; Wee et al., 2008; Rudoy et al., 2009; Forget et al., 2010; Verplaetse et al., 2012). Importantly, ICV injection of NE increases fos expression in the BNST (Brown et al., 2011) and β-AR antagonists microinjected into the extended amygdala can block stress-induced reinstatement (Leri et al., 2002) suggesting that dysfunction of NE systems in the extended amygdala is likely a key factor in enhanced drug-seeking following stress.

### Central amygdala neurocircuitry in addiction

The central amygdala (CeA) appears to contribute to the use of a number of different drugs. Acute and chronic alcohol/drug exposures and withdrawal increase CRF biosynthesis in the CeA (Merlo et al., 1995; Rodriguez de et al., 1997; Richter and Weiss, 1999; Maj et al., 2003; George et al., 2007; Zorrilla et al., 2012) and the CeA sends a CRF-containing projection to the BNST that is critical for stress-induced reinstatement (Erb et al., 2001). Therefore, an understanding of drug/alcohol interactions with CeA CRF neurocircuitry may provide an insight into an important interface between stress and addiction A series of studies have shown that EtOH enhances GABAergic neurotransmission in the CeA via a CRF type 1 receptor (CRFR1)-dependent mechanism (Roberto et al., 2003, 2010; Nie et al., 2009). Mice exposed to chronic intermittent ethanol (CIE) exhibit higher levels of EtOH drinking, increased GABA release, and heightened CeA CRFR1 sensitivity during withdrawal, suggesting a key role of CRF-GABA interaction in the CeA in the development of EtOH dependence (Roberto et al., 2004, 2010). Furthermore, treating mice with CRFR1 antagonists blocked the ability of CIE to increase alcohol drinking (Roberto et al., 2010). CIE-induced increases in alcohol self-administration are also blocked by an intra-CeA microinjection of a non-selective CRFR antagonist (Funk et al., 2006a). CeA CRF neurocircuitry is also activated during binge-like EtOH self-administration prior to the development of dependence and binge-like EtOH consumption can be reduced by intra-CeA microinjections of CRFR1 antagonists (Lowery-Gionta et al., 2012). Since CRFR1 antagonists can block stress-induced increases in EtOH self-administration (Hansson et al., 2006; Marinelli et al., 2007; Lowery et al., 2008), these findings indicate that changes in CeA CRF signaling may play an important role in the development and maintenance of EtOH addiction and in relapse.

In addition to its effects on CeA GABAergic neurotransmission and its functional role in EtOH induced alterations to CeA activity, CRFR1 can also enhance CeA glutamatergic neurotransmission. CRFR1 activation increases glutamate release from specific presynaptic sources in the CeA (Liu et al., 2004; Silberman and Winder, 2013) and can induce long-term potentiation of the BLA-CeA pathway (Fu et al., 2007). This effect can be manipulated by chronic drug exposures as withdrawal from chronic intermittent cocaine can enhance CRFR1 induced long-term potentiation of CeA synaptic transmission (Fu et al., 2007), suggesting that CeA CRF signaling is important for cocaine related behaviors and may play an important role in the development of cocaine addiction. Blockade of CeA CRFR1 can also attenuate dysphoria associated with nicotine withdrawal (Bruijnzeel et al., 2012). These findings suggest that changes in CeA CRF neurotransmission may play a role in addiction to multiple drug types. However, although CRF-producing neurons do exist in the CeA, it is not yet clear if these neurons are the source of extracellular CRF in the CeA as our recent studies suggests that CRF neurons in the CeA may be predominantly projection type (Silberman et al., 2013). Indeed, some evidence indicates that other brain regions may be the major source of extracellular CRF in the CeA (Uryu et al., 1992). It is also not yet clear how alcohol/drugs might alter the activity of CeA CRF neurons that project to the BNST. Future research will be needed to determine how CeA CRF signaling to the BNST is altered by chronic alcohol or drug exposure that may make them more sensitive to stress to promote CRF release in the BNST to initiate reinstatement.

### Bed nucleus of the stria terminalis neurocircuitry in stress-induced reinstatement

Alcohol and other drugs of abuse can also modulate CRF activity in the BNST. Protracted withdrawal from cocaine, heroin, and alcohol can result in a dysregulation of the intrinsic excitability of some BNST neurons via a CRF-mediated mechanism (Francesconi et al., 2009), suggesting that repeated activation of BNST CRF receptors likely plays a critical role in the development of drug-withdrawal symptomology. Furthermore, microinjections of CRFR1 antagonists into the BNST can block stress-induced reinstatement of drug-seeking (Erb and Stewart, 1999; Erb et al., 2001) while microinjections of CRF into the BNST can drive reinstatement for drug-seeking (Erb and Stewart, 1999). Together, these findings suggest that CRFR1 within the BNST is a critical component of stress-induced reinstatement behaviors.

While the above studies have shown a clear role of BNST CRF signaling in stress-induced reinstatement of cocaine seeking, it less clear what role CRF signaling in the BNST plays in alcohol addiction. For instance, although intra-CeA injections of CRF antagonists post CIE can block CIE-induced increases in EtOH self-administration, post-CIE intra-BNST injections of the same antagonist does not block enhanced drinking (Funk et al., 2006a). However, a series of studies indicate that BNST CRF signaling becomes enhanced during exposure to stressors that elicit reinstatement to ethanol seeking (Le et al., 2000; Funk et al., 2006b). Interestingly, cycles of stressors can substitute for cycles of intermittent EtOH exposures to increase withdrawal-induced anxiety, an effect that is also CRF receptor dependent (Breese et al., 2004). Furthermore, recent studies indicate that intra-BNST injections of CRF before ethanol exposure sensitized ethanol-withdrawal-induced anxiety while intra-BNST CRFR1 antagonist injections prior to stress blocked increases of anxiety-like behavior during ethanol withdrawal (Huang et al., 2010). Therefore, it is likely that the combination of repeated EtOH exposure and stressors (environmental stress or drug-withdrawal stress) sensitizes BNST CRF activity to promote anxiety-like behaviors in withdrawal. This sensitized BNST CRF activity may increase the likelihood of stress-induced reinstatement of ethanol and other drugs of abuse.

## Mechanisms of NE/CRF Interactions in Stress-Induced Reinstatement

Together, the findings reviewed above indicate that both NE and CRF in the extended amygdala are key components of both acute drug-withdrawal syndromes and reinstatement. Although we now have a better understanding of the neurocircuitry and neurotransmitter systems involved in stress-induced reinstatement, it is still unclear how chronic exposure to drugs modulates NE/CRF-related neurocircuitry in the extended amygdala to sensitize stress pathways and precipitate reinstatement. For these reasons, our lab and others have recently focused on this neurocircuitry to elucidate the major neuronal mechanisms involved in enhanced stress sensitivity following chronic drug exposure and role of this circuitry in the addiction process.

### NE/CRF interactions in the BNST promote reinstatement to drug seeking

While the work described in the previous section indicates an important role of NE and CRF signaling in modulation of BNST activity in stress-induced reinstatement behaviors, the mechanisms by which stress-related signaling modulates extended amygdala activity and how this modulated activity drives alcohol/drug seeking is not well understood. One clue as to the mechanism of BNST NE and CRF signaling is that pretreatment with a CRFR antagonist can block reinstating effects of AR stimulation while blockade of adrenergic signaling does not alter CRF-induced reinstatement (Brown et al., 2009). Given the likely role of β-AR receptors in the BNST in stress-induced reinstatement (Leri et al., 2002), these findings suggests that β-AR and CRF systems may interact in the BNST to initiate drug-seeking behavior following stress exposure and that β-ARs and CRFRs may work in a serial fashion to enhance BNST activity. To confirm this mechanism, our lab examined the role of β-ARs and CRFRs on glutamatergic transmission in the BNST (Nobis et al., 2011). In these studies, the β-AR agonist, isoproterenol, and CRF increased the frequency of spontaneous glutamatergic neurotransmission in the BNST. Interestingly, the effect of both drugs was blocked by pretreatment with a CRFR1 antagonist. The effects of CRF and isoproterenol were occluded during acute withdrawal from chronic cocaine exposure, suggesting that serial NE-CRF signaling in the BNST is engaged *in vivo* during drug exposures (Nobis et al., 2011).

### Potential role for CRF-producing neurons within the BNST in stress-induced reinstatement

While it has been established that elevated CRF levels in the BNST are important for stress-induced reinstatement, one remaining question is the source of elevated extracellular CRF in the BNST in response to stress exposure. CRF could be released from local neuronal sources, from extrinsic CRF projections from the CeA, or both (Veinante et al., 1997; Erb et al., 2001). To further explore this question, we hypothesized that if β-ARs enhance BNST CRF levels by modulating the activity of local CRF neurons, then isoproterenol would be expected to alter the activity of BNST neurons that produce CRF. On the other hand, if β-AR activation resulted in increased CRF from CeA sources, then the activity of BNST CRF neurons might not be altered by isoproterenol. To test this hypothesis, we recorded the activity of CRF-producing neurons in the BNST in a novel CRF-reporter mouse line (Silberman et al., 2013). To develop this line, we crossed two commercially available mouse lines from Jackson Laboratories, the *CRF-ires-cre* (strain B6(Cg)-Crhtm1(cre)Zjh/J) line and the ROSA-tomato [strain B6.Cg-t(ROSA)26Sor < tm14(CAG-tdTomato)Hze > /J] line. Crossing these two lines of mice resulted in offspring where a red fluorescent protein (*tomato)* was targeted to *cre* containing neurons, which in this case were neurons that produced *cre* under the control of the endogenous *Crf* promoter/enhancer elements (CRF-*tomato* mice). The *CRF-tomato* mice were found to have high levels of *tomato* expression in brain areas known to be dense in CRF-producing neurons, like the paraventricular nucleus of the hypothalamus, the CeA, and the BNST, while brain regions that are known to have little CRF-producing neurons, like the cortex and striatum, were shown to have sparse *tomato* expression.

We then preformed whole-cell patch clamp electrophysiology experiments on CRF-*tomato* neurons in the BNST. These studies indicate that there are several different subtypes of BNST CRF neurons based on electrophysiological characteristics. Three of the subtypes were similar to those previous shown to exist in the rat BNST (Hammack et al., 2007) while the two remaining subtypes have not previously been characterized. Research is currently ongoing in our lab to determine if distinct CRF neuronal subtypes play dissociable roles in BNST-mediated behaviors and if they are can be distinguished based on their projection targets or other neurochemical markers.

**Figure 1:**
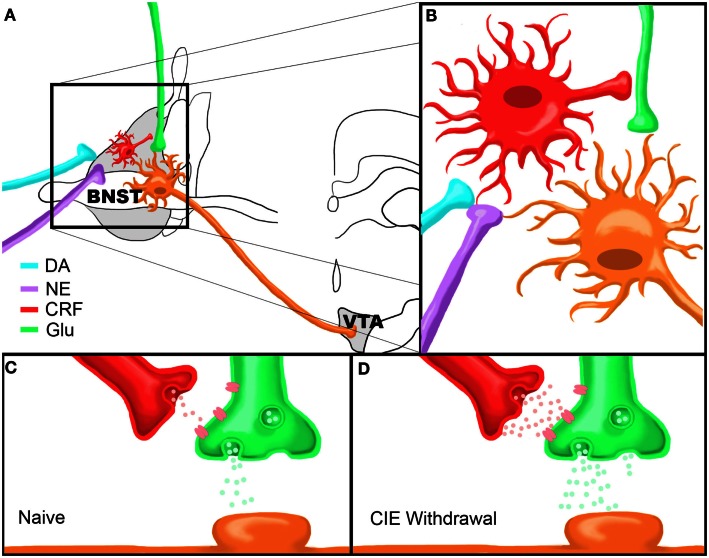
**Model of Chronic Intermittent Ethanol-Withdrawal Modulation of BNST CRF Circuitry. (A)** Dopamine and norepinephrine afferents synapse onto CRF-producing neurons in the BNST which in turn influence neurotransmitter release from glutamatergic afferents onto BNST neurons projecting to the VTA. **(B)** Close up view of proposed neurocircuitry described in **(A)**. **(C,D)** Model of CRF modulation of glutamatergic transmission onto a VTA-projecting BNST neuron in a drug-naïve state **(C)** or during acute ethanol withdrawal following CIE **(D)**. Note that there are higher levels of CRF and glutamate release during withdrawal compared to the drug-naïve state. Figure reprinted from (Silberman et al., 2013).

Regardless of these characteristic differences in CRF neuron subtypes, isoproterenol application resulted in a significant depolarization of BNST CRF neurons, an effect that was significantly correlated with increased input resistance. These data suggest a role of β-ARs in the direct depolarization of BNST CRF neurons through closure of a leak or voltage-gated channel. Such a depolarization could increase release of CRF from these neurons, although this has yet to be directly tested. Together, these data suggest that stress-induced increases in NE signaling in the BNST leads to enhanced local CRF neuron activity in the BNST which likely leads to enhanced CRF release. Enhanced extracellular CRF levels in the BNST in turn leads to enhanced glutamatergic activity in the BNST and thus increased BNST excitation (see summary Figure 1). This enhanced level of BNST CRF may be further modulated by CRF afferents from the CeA (Erb et al., 2001). Overall, CRF-mediated enhancement of excitatory drive in the BNST is likely a key participant in stress-induced reinstatement. The following section will further describe this proposed BNST neurocircuit and its sensitivity to drug-related permutations as a critical factor precipitating reinstatement to drug-seeking behaviors following withdrawal.

## Potential Role of BNST Projections to the VTA in Stress-Induced Reinstatement

Although the above described studies show a clear role for NE/CRF interactions in enhancing BNST excitability, it is not clear how enhanced BNST excitability leads to increased drug-seeking behavior following stress. As mentioned earlier, mesolimbic circuit activation is a critical component of drug-seeking behavior in all types of reinstatement models. Therefore, it is hypothesized that BNST afferents to the VTA may be an important pathway in initiation of drug-seeking behaviors following stress. The following sections will explore this possibility.

### Neuroanatomical and functional evidence for BNST-VTA circuitry in drug-seeking behaviors

A series of neuroanatomical studies showed that the BNST sends a dense set of projections to the VTA (Georges and Aston-Jones, 2001, 2002; Dong and Swanson, 2004, 2006a,b). Disconnection of this pathway reduces cocaine preference (Sartor and Aston-Jones, 2012) and BNST neurons projecting to the VTA become activated during reinstatement to cocaine seeking (Mahler and Aston-Jones, 2012), suggesting BNST projections to the VTA are important in multiple drug-related behaviors such as preference and drug seeking during reinstatement. Initial *in vivo* electrophysiology studies showed that electrical and pharmacological stimulation of the BNST can elicit increased firing of putative DA neurons in the VTA (Georges and Aston-Jones, 2001). This pathway was further characterized showing that antagonism of glutamatergic receptors in the VTA can block BNST stimulation mediated enhancement of VTA DA neuron firing while having minimal effects on putative VTA GABA neuron firing (Georges and Aston-Jones, 2002). Together, these anatomical and electrophysiology studies suggest that the BNST may regulate the activity of the VTA DA neurons during reinstatement.

More recent studies using optogenetic strategies suggest that parallel circuitry in the BNST can mediate distinct aspects of anxiety-like behaviors (Kim et al., 2013). These studies show that selective inactivation of cells in the region of the oval subnucleus of the dorsal BNST (ovBNST) is correlated to a reduction in anxiety-like behaviors and that ovBNST neurons inhibit the activity of the anterodorsal subregion of the BNST (adBNST). These studies further show that the adBNST contains neurons that project to the VTA, parabrachial nucleus, and lateral hypothalamus and that selective stimulation of these pathways may promote different aspect of anxiolysis, as measured by increased open arm time in an elevated plus maze and reduction in respiratory rates. Our recent evidence further suggests that these divergent projections likely arise from distinct subpopulations of neurons in the adBNST (Silberman et al., 2013). Kim et al. (2013) propose this arrangement of BNST neuronal signaling may facilitate modular circuit adaptations in response to environmental stimuli by independent tuning of divergent projection neuron populations. Especially relevant to this review, optogenetic stimulation of adBNST terminals in the VTA can elicit realtime place preference, suggesting that increased activity of certain BNST projection neurons are critical for regulation of VTA-mediated reward behavior (Jennings et al., 2013).

While the BNST contains multiple subnuclei and a variety of neuronal cell types based on immunohistochemical and electrophysiological characteristics (Egli and Winder, 2003; Dumont and Williams, 2004; Hammack et al., 2007; Kash et al., 2008), studies indicate that BNST neurons that project to the VTA may be sensitive to modulation by drugs of abuse (Dumont et al., 2008). Interestingly, more recent work has shown that BNST neurons that project to the VTA are more likely to become activated following a stressor than other BNST neurons (Briand et al., 2010). Together, these findings suggest that certain subpopulations of BNST neurons, i.e., VTA-projecting neurons, are particularly important to enhanced drug seeking following stress exposures.

### CRFR1 mediates ethanol-withdrawal-induced increases in glutamatergic transmission onto BNST neurons projecting to the VTA

In combination with previous evidence of the importance of BNST CRF signaling to stress-induced reinstatement, we hypothesized that CRF modulation of BNST neurons projecting to the VTA may be uniquely sensitive to drug-induced alterations in excitability. To test this hypothesis we have recently performed a series of experiments to determine the effect of CRF on glutamatergic transmission onto VTA-projecting BNST neurons and determine whether chronic drug exposures can modulate this system. VTA-projecting BNST neurons were identified by microinjecting retrograde fluorescent microspheres into the VTA and labeled neurons in the BNST were recorded using whole-cell electrophysiology methods (Silberman et al., 2013). In these studies, we showed that CRF, via activation of CRFR1, can enhance glutamate release onto BNST neurons projecting to the VTA. Combined with our data showing that β-AR activation depolarizes BNST CRF neurons, the above findings indicate that stress, via release of NE in the BNST, can increase BNST CRF activity to, in turn, increase glutamatergic signaling onto VTA-projecting BNST neurons (Figures 1A,B).

We then tested whether this pathway is modulated by abused drugs by exposing VTA-retrograde tracer mice to the CIE vapor exposure paradigm (CIE). This repeated ethanol exposure/withdrawal paradigm has been shown to increase anxiety-like behaviors during withdrawal (Kash et al., 2009) and increase voluntary ethanol drinking post-withdrawal (Becker and Lopez, 2004), suggesting that this paradigm is an important tool in assessing neurobiological changes in negative reinforcement pathways, such as the BNST, following drug exposure. Interestingly, we found that basal glutamatergic tone was increased in excitatory synapses that regulate VTA-projecting BNST neurons during the acute withdrawal phase after a 2 week CIE cycle. Also, from this enhanced basal glutamatergic tone, exogenous application of CRF could no longer enhance glutamatergic transmission as it could in drug-naïve or sham exposed mice. This functional occlusion of exogenous CRF suggests that CRF receptors may already be maximally active during acute drug-withdrawal time points, perhaps due to highly elevated extracellular CRF levels and sensitize BNST CRF circuitry. This may be one reason why post-CIE CRFR1 antagonist injections into the BNST do not block CIE-induced increases in ethanol self-administration (Funk et al., 2006a) and suggests that CRFR1 antagonist treatment prior to CIE may normalize BNST CRF circuitry during acute ethanol withdrawal. To examine this hypothesis, we exposed a second cohort of VTA-tracer mice to CIE with the inclusion of daily injections of a CRFR1 antagonist prior to ethanol vapor exposure. Pretreatment with a CRFR1 antagonist completely abolished the effects of CIE on increasing basal glutamatergic function during acute withdrawal timepoints. Together, these findings indicate that CIE modulates BNST CRF neurocircuitry *in vivo* and that this neurocircuit becomes hyperactive during CIE withdrawal (Figures 1C,D). An important caveat to these findings is that the role of BNST CRF sensitivity has mainly been examined during acute withdrawal phases and has provided potentially conflicting results. It will be important in future studies to examine the mechanisms by which sensitized BNST CRF circuitry may promote increased stress-induced drug-seeking behavior during later time points in extended withdrawal.

Although more work will be needed to conclusively show a role of this circuit in reinstatement behaviors, the recruitment of the catecholamine-CRF-glutamate circuit in the BNST to drive increased VTA activity is one promising mechanism by which stress can enhance drug seeking in reinstatement models. Interestingly, while the above described studies focused on the effect of ethanol on BNST CRF circuitry other work indicates that cocaine (Nobis et al., 2011) and opiates (Wang et al., 2006; Jaferi et al., 2009) may also stimulate BNST CRF neurocircuitry *in vivo*. Together, these findings suggest that modulation of BNST CRF may be a common pathway for stress-induced reinstatement for multiple classes of abused drugs. Therefore, therapeutics targeting this system may be useful for the effective long-term prevention of stress-induced relapse in addiction to many types of drugs.

## Proposed Model of BNST/VTA Circuitry in Stress-Induced Reinstatement

The studies described above suggest a critical role of increased activity of BNST neurons that project to the VTA in the neurophysiological response to stress and drug addiction. However, the mechanism by which activation of BNST projection neurons may modulate VTA activity is not clear.

### Multiple subtypes of BNST neurons project to the VTA

Some electrophysiological studies indicate that BNST projections to the VTA are likely to be glutamatergic, as they enhance VTA neuron firing (Georges and Aston-Jones, 2001, 2002). However, more recent work indicates that BNST projections to the VTA may be either glutamatergic or GABAergic (Jennings et al., 2013). Other recent studies utilizing fluorescence *in situ* hybridization and retrograde labeling techniques show that there are three types of VTA-projecting neurons in the BNST. The vast majority of these neurons (∼90%) are GAD+/VGlut− while other subtypes are VGlut2+/GAD− or VGlut3+/GAD+ (Kudo et al., 2012). This suggests that most VTA-projecting neurons in the BNST are GABAergic, while a minority of outputs may be glutamatergic or contain a mixture of transmitters. Our recent work shows that VTA-projecting BNST neurons can be divided into three classes based on electrophysiological responses to hyperpolarizing and depolarizing current injections (Silberman et al., 2013). Although it has yet to be tested, it is tempting to think that the differences in GAD and VGlut2/3 expression in BNST neuron subtypes may be related to differences in their electrophysiological firing properties. Still other studies suggest that at least some of the BNST neurons projecting to the VTA contain CRF (Rodaros et al., 2007). This is an important consideration as elevated CRF levels in the VTA can drive DA neuron activity after exposure to drugs of abuse by a number of mechanisms (Wise and Morales, 2010). Determining the contribution of these unique BNST projection neuron subtypes to stress-induced drug-seeking behavior may be useful in targeting future treatments for relapse prevention.

### Evidence for subtype specific BNST innervation of VTA GABA and VTA DA neurons

**Figure 2:**
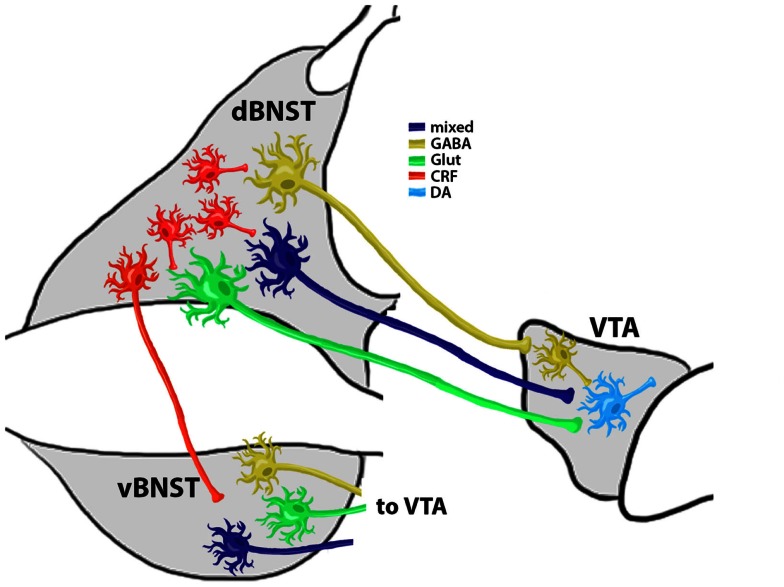
**Summary Model of Reinstatement Related BNST and VTA Connectivity**. CRF+ neurons modulate the activity of VTA-projecting BNST neurons. Evidence (Kudo et al., 2012) shows that at least three types of VTA-projecting neurons are located in the BNST: (1) a GABAergic projection (∼90% of all BNST projection neurons) that selectively innervates VTA GABA neurons to provide disinhibition of VTA DA neurons; (2) a glutamatergic (Glut) projection that selectively targets VTA DA neurons; and (3) a mixed GABA/Glut projection that also targets VTA DA neurons. These projection neuron populations may exist in both the dorsal and ventral BNST subregions (d and vBNST, respectively) and each projection pathway may have distinct and coordinated responses to chronic drug exposure, withdrawal, and reinstatement. Coordinated activity of dBNST and vBNST projection neurons is likely regulated by dBNST interneurons, of which CRF+ neurons may be a critical component. This local CRF neuron coordination of BNST activity might also be altered by chronic exposure and withdrawal and may be an important target for the prevention of relapse-like behaviors.

Overall these findings indicate that the BNST sends a mixture of neurotransmitters to the VTA. However, what is less clear is whether distinct types of BNST projection neurons synapse to different VTA neurons. Recent evidence indicates that selective optogenetic stimulation of VTA GABA neurons disrupts reward consumption (van Zessen et al., 2012) and increased conditioned place aversion (Tan et al., 2012). Furthermore, selective optogenetic stimulation of VTA DA neurons can enhance positive reinforcing actions in an operant food seeking task and can reactivate previously extinguished food seeking behavior in the absence of cues (Adamantidis et al., 2011). Interestingly, recent immunoelectron microscopy work indicates that vGLUT containing BNST projection neurons may selective target VTA DA neurons while GABAergic BNST projection neurons may specifically target GABA neurons in the VTA [(Kudo et al., 2012) although see also (Jennings et al., 2013)]. Together, these findings may indicate that enhanced activity of BNST projections to the VTA during reinstatement may stimulate VTA DA neurons via increasing local glutamatergic levels while at the same time disinhibiting VTA DA neuron firing by inhibiting local GABA release (see model, Figure 2). This may be one mechanism by which drug-withdrawal enhances burst firing of VTA DA neurons (Hopf et al., 2007), an effect that is important in drug-seeking behaviors (Wanat et al., 2009), and may be especially important in stress-induced reinstatement models.

The precise role of distinct VTA-projecting BNST neurons in reinstatement is not yet fully understood. For instance, although evidence suggests that BNST neurons that project to the VTA can be mainly GABAergic, but also glutamatergic or potentially both (Kudo et al., 2012), it is not clear if these pathways have an equal distribution of synaptic strength. Furthermore, some BNST projections to the VTA may contain CRF (Rodaros et al., 2007) but it is not clear which of the VTA-projecting neurons described by Kudo et al. or Jennings and Sparta et al. are also CRF positive. If so, this may suggest that a single population of VTA-projecting BNST neurons may have divergent modes of action in reinstatement related behaviors based on which neurotransmitter is released at specific time points relative to reinstatement trigger exposure. Lastly, most of the electrophysiology studies described in this review focused on neurocircuitry in the dorsal subregion of the BNST while most of the behavioral work has focused on activity of the ventral BNST subregion. This is an important consideration as the dorsal BNST, which has a high proportion of GABAergic interneurons, sends afferents to the ventral BNST, which has a higher proportion of projection neurons (Dong et al., 2001). This suggests that the dorsal BNST might coordinate overall BNST output via modulation of ventral BNST projection neurons, potentially via BNST CRF interneuron activity. It is not yet clear if interneurons or VTA-projecting neurons from the dorsal and ventral BNST are equally mutable to chronic drug exposures/withdrawal cycles. While more conclusive research will be needed to test these intriguing possibilities, these findings may indicate dissociable roles of BNST projection neuron subtypes in mediating various aspects of drug-seeking behavior during reinstatement that could potentially be targeted individually for pharmacotherapies for relapse prevention in the future.

## Potential Role of BNST CRF Signaling in Cue-Induced Reinstatement

### Evidence for direct and indirect dopaminergic activation of BNST in cue-induced reinstatement

In addition to its role in stress-induced reinstatement described above, recent evidence may suggest that BNST CRF neurocircuitry could also play a role in cue-induced reinstatement. BLA DA receptor activation is critical for cue-induced reinstatement (See et al., 2001) and DA can increase BLA activity, but only after chronic drug exposure (Li et al., 2011). Since the BLA sends direct projections to the BNST as well as via indirect projections through the CeA (Davis et al., 2010), DA induced activation of the BLA may enhance BNST excitability to precipitate reinstatement following a cue exposure. In addition, drugs of abuse and other rewarding stimuli can also directly increase extracellular DA levels in the BNST (Carboni et al., 2000; Park et al., 2012). Previous work in our lab shows that DA can enhance glutamate release in the BNST via activation of CRFR1 (Kash et al., 2008). This effect is further confirmed by our more recent work indicating that DA can depolarize BNST CRF neurons (Silberman et al., 2013). Together, these findings suggest both direct and indirect mechanisms for DA induced increases in BNST excitability and point to a potential role of BNST DA circuitry in cue-induced reinstatement via modulation of BNST CRF circuitry.

Importantly, behavioral evidence also shows a potential role for the BNST in cue-induced reinstatement models. For instance, recent findings indicate that pharmacological inactivation of the BNST can reduce cue-induced reinstatement (Buffalari and See, 2011). In addition, much like earlier studies showing selective increases in c-fos in VTA-projecting BNST neurons following stress-induced reinstatement, recent findings show that increased c-fos activation in VTA-projecting BNST neurons is correlated to enhanced cocaine-seeking following an exposure to a drug-associated cue (Mahler and Aston-Jones, 2012). Together with our electrophysiology data, these findings suggest that DA may increase extracellular CRF levels in the BNST via enhancing the activity of local BNST CRF neurons, which in turn increases glutamate release onto VTA-projecting BNST neurons, leading to increased VTA DA firing to reinstate drug-seeking behaviors.

### Evidence for convergence of cue-induced and stress-induced reinstatement pathways in the BNST

Interestingly, while clinical evidence shows that exposing recovering addicts to drug-associated cues results in enhanced feelings of craving, recent findings indicate that these same cues also increase feelings of negative affect (Fox et al., 2007). Therefore, drug-associated cues could act as a psychological stress by activating stress-related neurocircuitry. This suggests that drug-associated cues may concurrently increase both DA and NE signaling in these patients. Our data suggest that DA and NE can additively enhance BNST excitability (Nobis et al., 2011), suggesting a convergence of cue-induced (dopaminergic) and stress-induced (noradrenergic) reinstatement pathway influences on BNST excitability. Preclinical studies also suggest a link between cue and stress-induced reinstatement (Buffalari and See, 2009) suggesting that simultaneous exposure to drug-cues and stress can greatly increase the risk of relapse in recovering addicts. Together, these findings indicate that BNST CRF signaling is an important potential target for convergent influences of both cue and stress-induced reinstatement pathways.

## Summary and Potential Treatments

The findings reviewed here suggest that a catecholamine-CRF-glutamatergic signaling pathway in the BNST plays an important role in the reinstatement to drug-seeking behavior, an important animal model of relapse to alcohol/drug addiction. While this pathway is clearly important in stress-related behaviors, especially in stress-induced reinstatement, further studies suggests that this pathway may also be important in cue-induced reinstatement. Therefore, pharmacotherapies targeting this pathway may be useful in the prevention of relapse to both drug-associated cues and stressors. Unfortunately, relapse can be a life-long struggle in recovering addicts, which means that pharmacotherapies to prevent relapse likely need to be taken daily for extended periods of time. Therefore these therapies need to be well-tolerated and devoid of harsh side-effects. As described earlier, agonist therapies targeting the DA aspect of this pathway may be problematic from the side-effect standpoint due to effects on the cardiovascular system and abuse liability. DA antagonist therapies are also problematic for their potential for extra-pyramidal (Peacock et al., 1999) and anhedonic side effects (Stein, 2008). Recent studies have looked into the effect of β-AR antagonists to reduce the probability of relapse in the clinical population (Hughes et al., 2000; Kampman et al., 2001; Schwabe et al., 2011). Overall, these studies have shown β-AR antagonist to potentially be useful in the clinical setting, especially for reducing stress-induced changes in habitual behaviors and in those patients that have more severe withdrawal symptoms. However, it is unclear if treatment with β-AR antagonists would have an effect on cue-induced relapse.

Since DA and β-AR activation enhances BNST activity via CRFR1 activation, then CRFR1 antagonists might be a better alternative for the effective long-term prevention of both cue and stress-induced relapse. CRFR1 antagonists have been shown to reduce ethanol intake following withdrawal in a number of preclinical studies (Funk et al., 2007; Logrip et al., 2011). To date, there have been no studies examining the effectiveness of CRFR1 antagonists in relapse prevention in the clinical setting. However, this class of drugs has been studied in the clinical setting to treat anxiety disorders and other stress-related disorders. While these studies have shown limited effectiveness of CRFR1 antagonists in treating general anxiety disorder (Coric et al., 2010) or irritable bowel syndrome (Sweetser et al., 2009), these compounds can produce significant signal reductions in the amygdala during pain expectation in humans (Hubbard et al., 2011). These findings suggest that CRFR1 antagonists may be useful in reducing negative affect in response to specific psychological stimuli. Importantly, these drugs are very well tolerated in the above mentioned studies and have been shown to cause no significant side-effects (Kunzel et al., 2003; Schmidt et al., 2010). However, to date many CRF antagonists have been shown to have undesirable lipophilic or pharmacokinetic profiles limiting their bioavailability and efficacy in clinical trials (Zorrilla and Koob, 2010). CRF antagonists with better pharmacokinetics may prove useful in the treatment of addiction in the future through interference with the proposed BNST CRF reinstatement circuit described here. Overall, CRF circuitry within the BNST is a critical locus for interactions between stress and reward signaling in addiction and may be an important target requiring further study for the treatment of relapse and addiction.

## Conflict of Interest Statement

The authors declare that the research was conducted in the absence of any commercial or financial relationships that could be construed as a potential conflict of interest.
